# Relative dose intensity over the first four weeks of lenvatinib therapy is a factor of favorable response and overall survival in patients with unresectable hepatocellular carcinoma

**DOI:** 10.1371/journal.pone.0231828

**Published:** 2020-04-20

**Authors:** Sakura Kirino, Kaoru Tsuchiya, Masayuki Kurosaki, Shun Kaneko, Kento Inada, Koji Yamashita, Leona Osawa, Yuka Hayakawa, Shuhei Sekiguchi, Mao Okada, Wan Wang, Mayu Higuchi, Kenta Takaura, Chiaki Maeyashiki, Nobuharu Tamaki, Yutaka Yasui, Hiroyuki Nakanishi, Jun Itakura, Yuka Takahashi, Yasuhiro Asahina, Namiki Izumi

**Affiliations:** 1 Department of Gastroenterology and Hepatology, Musashino Red Cross Hospital, Tokyo, Japan; 2 Department of Gastroenterology and Hepatology, Tokyo Medical and Dental University, Tokyo, Japan; 3 Department of Liver Disease Control, Tokyo Medical and Dental University, Tokyo, Japan; Centre de Recherche en Cancerologie de Lyon, FRANCE

## Abstract

Lenvatinib is an approved first-line therapy for unresectable hepatocellular carcinoma (HCC), but the effect of dose modification on its efficacy is unclear. We analyzed the relationship between the relative dose intensity during the initial 4 weeks of therapy [4W-relative dose intensity (RDI)] and the efficacy of lenvatinib therapy in the real-world setting. A total of 48 consecutive patients with unresectable HCC who received lenvatinib therapy for more than 4 weeks were included. The 4W-RDI was calculated as the cumulative dose in the initial 4 weeks divided by the weight-based standard dose, and we evaluated its association with overall survival (OS) and best response by modified Response Evaluation Criteria in Solid Tumor (mRECIST). The baseline factors predicting high 4W-RDI were analyzed further. The median durations of follow-up and of therapy among the 48 participants were 7.6 and 6.6 months, respectively. The median OS was not reached. Drug interruption and/or dose reduction were necessary in 30 patients (62.5%) and the median 4W-RDI was 70% (range 22%–100%). Patients with 4W-RDI ≥70% had longer OS [hazard ratio (HR) 0.28, 95% confidential interval (CI):0.09–0.90, p = 0.03], and longer duration of lenvatinib therapy (HR 0.39, 95%CI:0.16–0.92, p = 0.03). Patients with 4W-RDI ≥70% showed higher disease control rate compared to those with 4W-RDI <70% (91.7% vs. 54.2%, p = 0.008). A baseline albumin level >3.4g/dL or ALBI score less than -2.171 were significantly associated with achieving 4W-RDI ≥70%. In conclusion, 4W-RDI of lenvatinib therapy is associated with favorable radiological response and longer OS.

## Introduction

Lenvatinib (LEN) is a tyrosine kinase inhibitor (TKI) which has been approved for unresectable hepatocellular carcinoma (HCC) as a 1st line treatment. This was based on confirmation of its non-inferiority versus sorafenib in the phase III REFLECT study [1]. The efficacy of lenvatinib has also been reported in several real-world practices [2–5]. However, an important drawback of TKI therapy is that some cases need drug interruptions or dose reductions at early phase of the treatment because of adverse events [1, 6–8]. Thus, cumulative doses taken by patients often differ from weight-based planned doses, and to date, the relation between dose intensity and treatment effects has not been fully verified. The relative dose intensity (RDI) is a tool for assessing the total dose of chemotherapy. It is defined as the actual dose received divided by the standard calculated dose during a set period [9, 10] and is reported as being associated with favorable treatment response to TKI therapies in various cancers [11–13].

In this study, we analyzed relation between RDI during the initial 4 weeks of therapy (4W-RDI) and the efficacy of LEN for unresectable HCC in a real-world setting.

## Methods

### Patient

A total of 60 patients with unresectable HCC who received LEN therapy at Musashino Red Cross Hospital from April 2018 to May 2019 were included. To evaluate effects of LEN therapy, 12 patients who could not continue LEN therapy for 4 weeks were excluded. Subsequently, 48 patients were enrolled in this study. Flow chart of the study is shown in S1 Fig. HCC was diagnosed by contrast enhanced computed tomography (CT) or magnetic resonance imaging (MRI) when tumors displayed vascular enhancement in the early phase and washout in the later phase according to guidelines from the American Association for the Study of Liver Diseases [14] and the Japan Society of Hepatology [15]. Tumor biopsy was used to diagnose tumors with nontypical imaging findings.

### Protocol of LEN therapy

The standard dose of LEN therapy was determined by body weight: patients weighing <60 kg were given 8 mg/day and those weighing ≥60 kg were given 12 mg/day. Starting with a reduced dose was permitted based on the patient’s condition and the preference of the attending physicians. Tumor assessment was done in accordance with the modified Response Evaluation Criteria in Solid Tumor (mRECIST), using dynamic CT within 4–8 weeks and every 8 weeks thereafter [16, 17]. During follow up, all patients were measured routine blood-chemistry and tumor markers testing every 2–4 weeks.

The albumin-bilirubin (ALBI) grade was used to assess hepatic reserve function. This score was calculated based on serum albumin and total bilirubin (T.Bil) values using the following formula: [ALBI score = (log10 T.Bil(μmol/L) × 0.66) + (albumin (g/L) × −0.085)]. Scores were then interpreted as follows: grade 1, if less than or equal to −2.60; grade 2, if greater than −2.60 but less than or equal to −1.39; and grade 3, if greater than −1.39 [18, 19].

Adverse events (AEs) were graded according to the CTCAE version 5.0. The LEN dose was reduced or treatment was interrupted for AEs of at least grade 2 or for AEs considered otherwise unacceptable. LEN treatment was interrupted until adverse events were improved to less than grade1 or got acceptable. Doses were reduced from 12mg to 8mg and 8mg to 4mg. Alternatively, maintenance of the same dose with planned drug withdrawal for 2 days/week, such as weekday (5days)-on and weekend (2days)-off schedule, were permitted based on the patient’s condition and the preference of the attending physicians.

### Calculation of RDI

RDI was defined as the actual dose divided by the standard dose [9–11]. To evaluate RDI of early phase of LEN therapy, the 4-week RDI (4W-RDI) was calculated as the cumulative dose within the initial 4 weeks of starting LEN treatment divided by the standard dose.

### Statistical analysis

Overall survival (OS) was calculated using the Kaplan–Meier method and difference was analyzed by the log-rank test. Statistical significance was defined as a *p* value of <0.05. The factors associated with OS were analyzed using the Cox-proportional hazard model, and the backward stepwise selection method was used for multivariate analysis. Associations between 4W-RDI and factors were evaluated with Fisher’s exact test and Mann–Whitney U-test. Correlations between two variables were tested using Pearson’s correlation analysis. All statistical analyses were performed with EZR (Saitama Medical Centre, Jichi Medical University, Shimotsuke, Japan), a graphical user interface for R version 3.2.2 (The R Foundation for Statistical Computing, Vienna, Austria) [20].

This study protocol conformed to the ethical guidelines in the Declaration of Helsinki and was approved by Musashino Red Cross Hospital Clinical Research Ethics Committee. Written informed consent was obtained from every patient.

## Results

### Baseline characteristics

Baseline characteristics of patients are shown in Table 1. Median age was 74 years. All patients had an Eastern Cooperative Oncology Group Performance Status Scale (ECOG PS) score of 0 (30 patients) or 1 (18 patients). 44 patients (91.7%) were Child Pugh grade A and 4 patients (8.3%) were Child Pugh grade B. 12 patients (25.5%) had macrovascular invasion (MVI). 30 patients (62.5%) were treated as 1^st^ line tyrosine kinase inhibitor. Patients treated as 2^nd^ line and 3^rd^ line were 4 (8.3%) and 14 (29%), respectively.

**Table 1:** Baseline characteristics.

Age, years median (range)	74(52–92)
Male gender, n(%)	42(87.5)
Body weight(Kg), median (range)	60.0(30.3–101)
Etiology: HCV/HBV/Alcohol /Others, n(%)	26(54.2)/9(18.8)/5(10.4)/8(16.7)
PS (ECOG) 0/1, n(%)	30(62.5)/18(37.5)
Child Pugh Grade A/B, n(%)	44(91.7)/4(8.3)
ALBI Grade: 1 / 2 /3, n(%)	13(27.0)/34(70.9)/1(2.1)
AFP(ng/ml), n(%)	104.9(1.60–115112)
BCLC Stage B/C, n(%)	17(35.4)/31(64.6)
MVI, n(%)	12(25.5)
Extrahepatic spread, n(%)	21(43.8)
Clinical course: 1^st^ line/ 2^nd^ line/ 3^rd^ line, n(%)	30(62.5)/4(8.3)/14(29.2)
Initial dose: 4mg/8mg/12mg, n(%)	9(18.8)/21(43.8)/18(37.5)
Follow-up duration (Month), median (range)	7.6(1.8–15.7)
4W-RDI(%), median (range)	70(22–100)

AFP: α-Fetoprotein; BCLC: Barcelona Clinical Liver Cancer; HCV: hepatitis C virus; HBV: hepatitis B virus; MVI: macrovascular invasion; PS: performance status; 4W-RDI: 4 week relative dose intensity.

### Efficacy of LEN therapy

**Fig 1:**
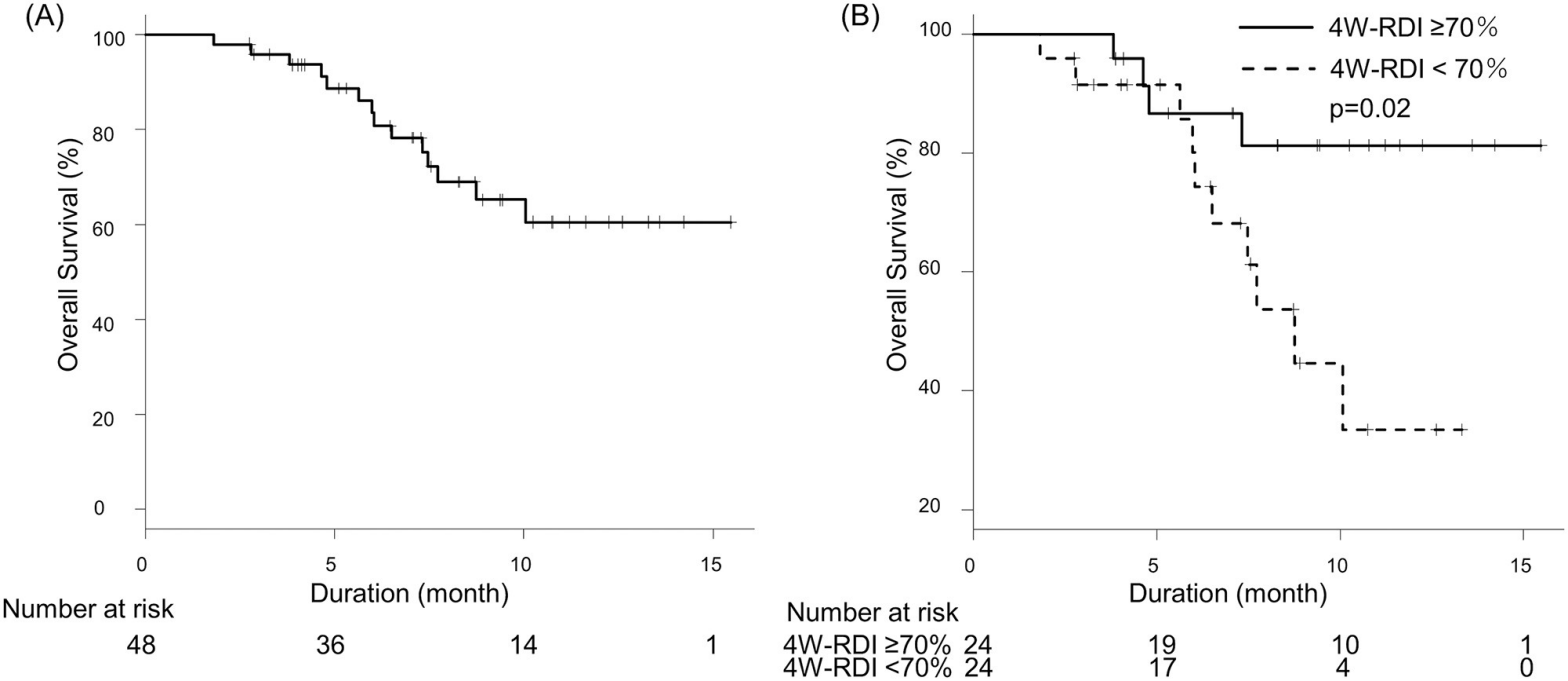
Overall survival. (A) OS of all patients. Median duration of follow up was 7.6 months, and median OS was not reached. (B) OS stratified by 4W-RDI. Patients with 4W-RDI ≥70% showed significantly longer OS compared to those with 4W-RDI <70% (p = 0.02). Median OS was not reached in patients with 4W-RDI ≥70% and 8.7 months in 4W-RDI <70%.

**Fig 2:**
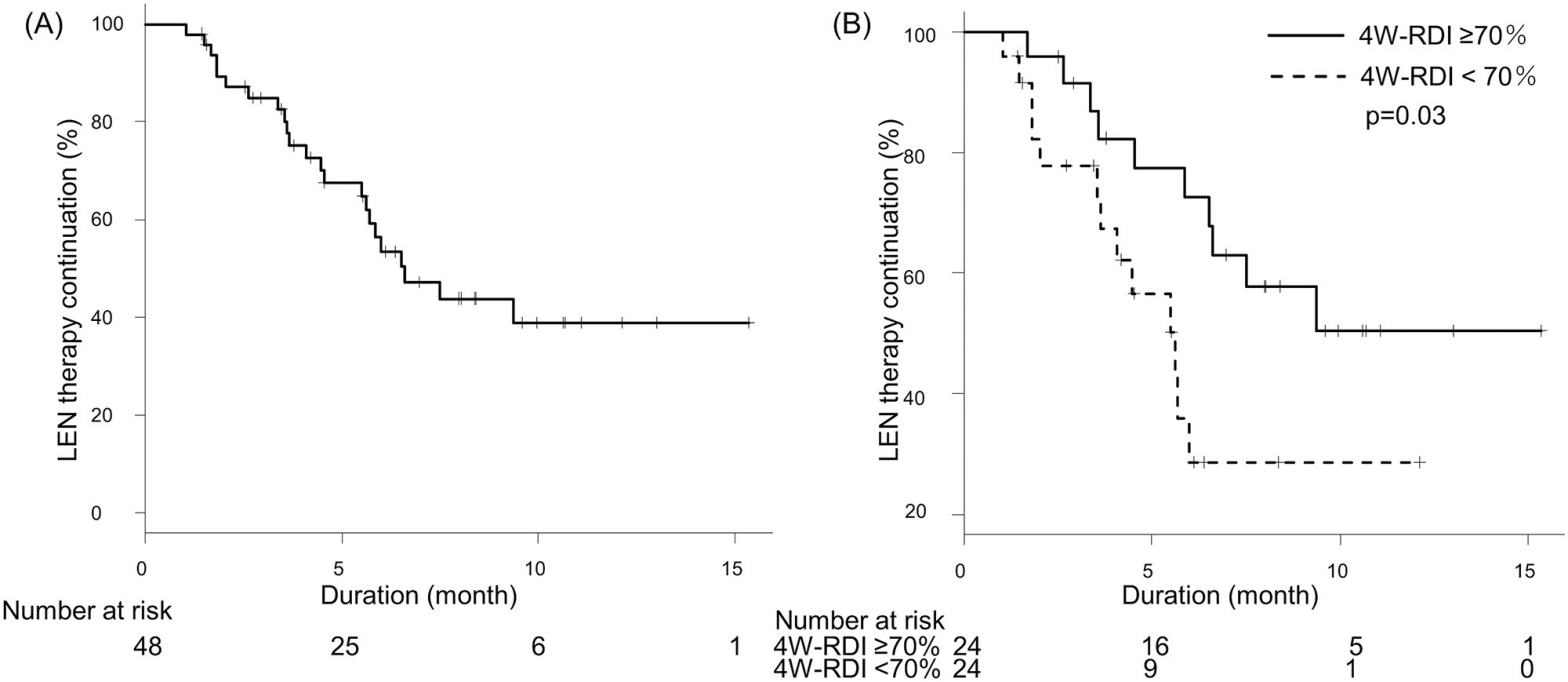
Time to discontinuation of lenvatinib therapy. (A) Time to discontinuation of lenvatinib therapy of all patients. Median duration of LEN therapy was 6.6 months. (B) Time to discontinuation of lenvatinib therapy stratified by 4W-RDI. Patients with 4W-RDI ≥70% were able to continue LEN treatment longer (p = 0.03). Median duration of LEN therapy was 9.5 months in patients with 4W-RDI ≥70% and 5.6 months in 4W-RDI <70%.

OS and duration of LEN therapy were shown in Figs 1A and 2A. Median OS was not reached and median duration of LEN therapy was 6.6 months. Median progression free survival (PFS) was 6.0 months. Radiological best response rates for complete response (CR), partial response (PR), stable disease (SD) and progression disease (PD) were 6.3%, 33.3%, 35.4% and 25.0%, respectively. Overall response rate (ORR) and disease control rate (DCR) were 39.6% and 75.0%, respectively.

### Distribution of 4W-RDI

**Fig 3:**
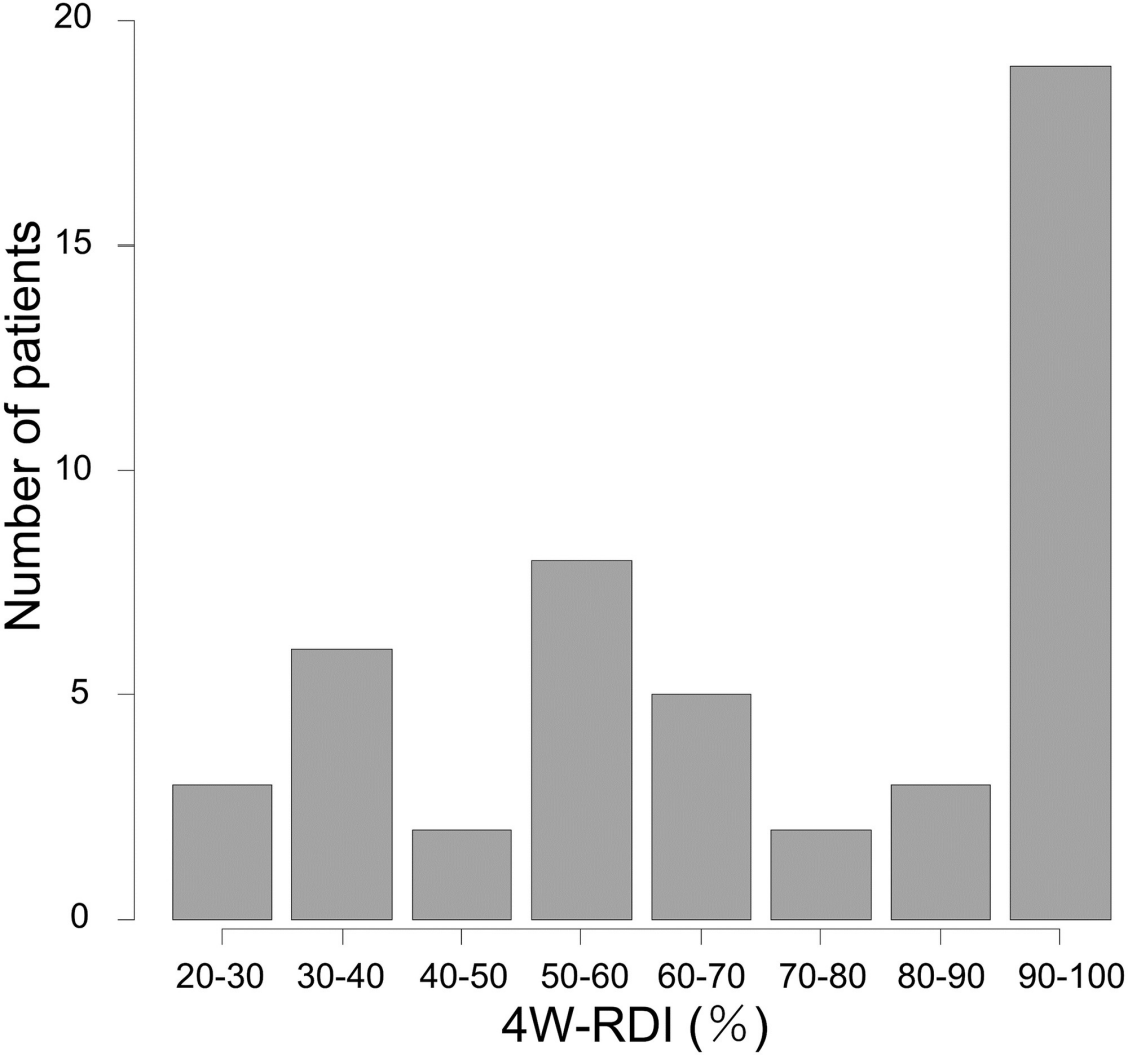
Distributions of patients by 4W-RDI. Patient distribution from low to high 4W-RDI categorized in 10% increment (median = 70%).

There were variations of 4W-RDI among cases, as shown by the distribution in Fig 3. Only 18 patients (37.5%) maintained 4W-RDI of 100%, and 30 patients (62.5%) required drug interruption and/or dose reduction in the 4weeks after administration. 16 patients (33.3%) had initial dose reduction. The reasons of initial dose reduction were impaired renal function (37.5%), Child Pugh B (18.8%), 40kg or lower body weight (12.5%), under anticoagulation therapy for pulmonary thrombosis (12.5%), decrease of appetite and depression (6.2%), past history of severe hand-foot syndrome by sorafenib treatment (6.2%), 50% or higher liver occupation of tumor (6.2%), respectively. Among them no patient was able to increase the dose. 15 patients (31.3%) needed drug interruption, and reasons of initial dose reductions were old age (37.5%), low renal function (37.5%), after treatments of infection (12.5%), thrombosis (6.2%), low platelet counts (6.2%), decrease of appetite (6.2%), past history of thyroid dysfunction (6.2%) and low body weight (6.2%), respectively. Reasons of dose reductions were fatigue (25%), proteinuria (20%), AST/ALT increase (15%), NH3 increase (10%), renal dysfunction (10%), hand-foot-syndrome (5%), nausea (5%), decreased appetite (15%), body weight decrease (5%), respectively. 20 patients (41.7%) needed dose reduction and reasons of dose interruptions were fatigue (40%), AST/ALT increase (26.7%), renal dysfunction (20%), NH3 increase (6.7%), nausea (6.7%), decreased appetite (6.7%), infection (6.7%), respectively. Median duration of dose interruptions was 6 (2–17) days Median 4W-RDI was 70% because of these modifications.

### Relation between 4W-RDI and OS

The relation between 4W-RDI and OS is shown with various cutoff values of 4W-RDI at 10% intervals in Table 2. When cutoff value was 70%, there was a statistically significant difference in OS [hazard ratio (HR) 0.28, 95% confidential interval (CI):0.09–0.90, p = 0.03]. Patients with 4W-RDI ≥70% showed significantly longer OS compared to those with 4W-RDI <70% (Fig 1B, p = 0.02). Median OS was not reached in patients with 4W-RDI ≥70% and 8.7 months in 4W-RDI <70%.

**Table 2:** Comparison of OS according to 4W-RDI cutoff value.

4W-RDI cutoff	Patient number	OS		
		HR	95%CI	p Value
≥40%/<40%	39/9	1.03	0.23–4.64	0.96
≥50%/<50%	34/14	0.91	0.25–3.30	0.89
≥60%/<60%	29/19	0.61	0.21–1.78	0.37
≥70%/<70%	24/24	0.28	0.09–0.90	0.03
≥80%/<80%	22/26	0.32	0.10–1.06	0.06
≥90%/<90%	19/29	0.49	0.15–1.57	0.23

CI: confidence interval; HR: hazard ratio; OS: overall survival; 4W-RDI: 4 week relative dose intensity.

In the univariable analysis, 4W-RDI ≥70% was significantly associated with longer survival than 4W-RDI <70%, whereas there were no significant differences by age (HR 0.99, 95%CI:0.94–1.04, p = 0.66), gender (HR 0.63, 95%CI:0.08–5.07, p = 0.67), previous TKI experience (HR 0.58, 95%CI:0.20–1.65, p = 0.30), α-Fetoprotein (AFP) level (HR 1.00, 95%CI:0.99–1.00, p = 0.61), ALBI score (HR 1.80, 95%CI:0.56–5.81, p = 0.33) and BCLC Stage C (HR 1.34, 95%CI:0.42–4.30, p = 0.62) (S1 Table).

In the multivariable analysis, the 4W-RDI ≥70% remained an independent prognostic factor of lenvatinib therapy (HR 0.3, 95%CI:0.09–0.96, p = 0.04) after adjusting for variables based on existing knowledge of prognostic factors of unresectable HCC such as age, gender, ALBI score, and BCLC stage (S1 Table).

### 4W-RDI and duration of LEN therapy

The relation between 4W-RDI and duration of LEN therapy is shown in Fig 2B. Patients with 4W-RDI ≥70% were able to continue LEN treatment longer beyond the initial 4 weeks (p = 0.03). In the univariable analysis, 4W-RDI ≥70% was a factor of long duration of lenvatinib therapy (HR 0.39, 95%CI:0.16–0.92, p = 0.03). Median duration of LEN therapy was 9.5 and 5.6 months in patients with 4W-RDIs of ≥70% and <70%, respectively.

### Relation between 4W-RDI and radiological best response

Radiological best response rates stratified by 4W-RDI are shown in Table 3. DCR of patients with 4W-RDI ≥70% was significantly high compared to 4W-RDI <70% (95.8% vs 54.2% p = 0.002). Overall response rate (ORR) of 4W-RDI ≥70% patients and 4W-RDI <70% patients were 54.2% and 25.0%, respectively (p = 0.08).

**Table 3:** Radiological responses to lenvatinib therapy by 4W-RDI.

	4W-RDI ≥ 70%	4W-RDI <70%	p value
CR	2(8.3%)	1(4.2%)	
PR	11(45.8%)	5(20.8%)	
SD	10(41.7%)	7(29.2%)	
PD	1(4.2%)	11(45.8%)	
ORR	13(54.2%)	6(25.0%)	0.08
DCR	23(95.8%)	13(54.2%)	0.002

CR: complete response; DCR: disease control rate; ORR: overall response rate; PD: progressive disease; PR: partial response; SD: stable disease; 4W-RDI: 4 week relative dose intensity.

### Baseline factors associated with high 4W-RDI

**Fig 4:**
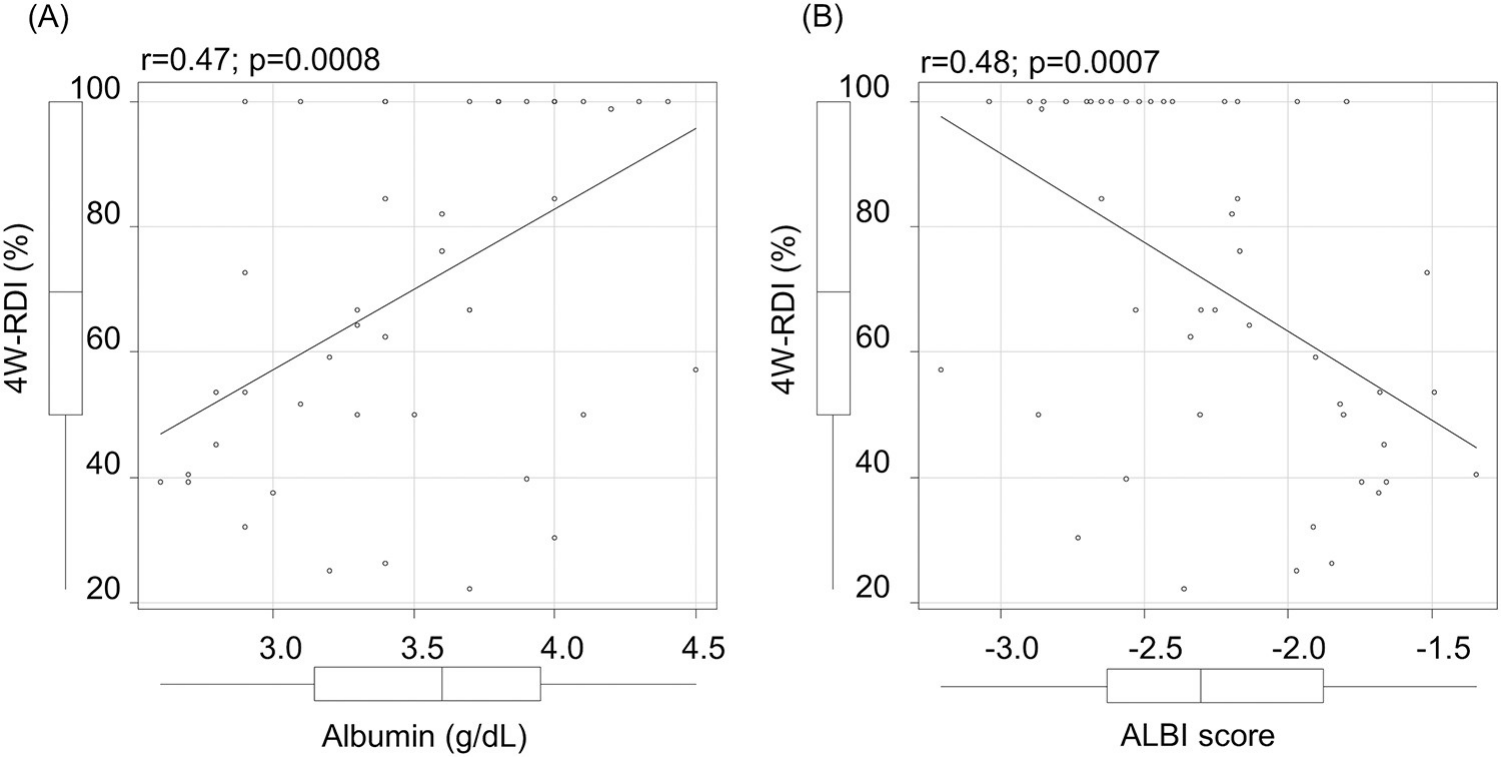
Correlation between 4W-RDI and baseline albumin or ALBI. (A) Correlation between 4W-RDI and baseline albumin level. (B) Correlation between 4W-RDI and baseline ALBI score.

Baseline characteristics of patients with 4W-RDI ≥70% and <70% are shown in Table 4. Mean ALBI scores of patients with 4W-RDI ≥70% and those with 4W-RDI <70% were -2.52 and -1.94, respectively and showed statistically significant difference (p = 0.006). Mean albumin level, which is a component of ALBI score were 3.8g/dL and 3.3g/dL, respectively (p = 0.003). A Pearson correlation analysis between 4W-RDI and ALBI score or 4W-RDI and albumin level revealed a significant correlation (ALBI score r = 0.48; p = 0.0007, albumin r = 0.47; p = 0.0008, respectively) (Fig 4).

**Table 4:** Factors associated with 4W-RDI≥70%.

	4W-RDI ≥ 70%	4W-RDI <70%	p value
Age, years, median (range)	75(54–92)	76(52–87)	0.77
Male gender, n(%)	23 (95.8)	19 (79.2)	0.19
Body weight(Kg), median (range)	65.1 (49.3–94.1)	58.2 (30.3–101.0)	0.05
ALBI score, median (range)	-2.52 (-1.52 - -3.04)	-1.94 (-1.35 - -3.21)	0.006
Albumin (g/dL), median (range)	3.8 (2.9–4.4)	3.3 (2.6–4.5)	0.003
T.Bil (mg/dL), median (range)	0.7 (0.4–1.9)	0.75 (0.4–2.2)	0.90
AFP (ng/ml), n (%)	38.4(1.6–37334)	227.1(2.1–115112)	0.61
TKI naïve, n (%)	17 (70.8)	13 (54.2)	0.37
MVI, n (%)	5(20.8)	7(30.4)	0.52
BCLC stage C, n (%)	17 (70.8)	14 (58.3)	0.55
Total AEs of grade 3/4, n (%)	4 (16.7)	14 (58.3)	0.01

AEs: adverse events; BCLC: Barcelona Clinical Liver Cancer; MVI: macrovascular invasion; TKI: tyrosin kinase inhibitor; 4W-RDI: 4 week relative dose intensity.

For prediction of 4W-RDI ≥70% by baseline albumin, the AUROC (area under the receiver operating characteristic) was 0.76 (95%CI:0.61–0.90) and the best cutoff values was 3.4g/dL [sensitivity:83.3%, specificity:58.3%, PPV (positive predictive):66.7%, NPV (negative predictive value):77.8%]. By baseline ALBI score, AUROC was 0.73 (95%CI:0.58–0.88) and the best cutoff values was -2.171 (sensitivity:83.3%, specificity:58.3%, PPV66.7%, NPV:77.8%). Baseline albumin and ALBI score both showed high predictability for 4W-RDI ≥70%.

### Adverse events

Adverse events (AEs) that occurred in this study are shown in Table 5. Any grades of total AEs were observed in 47 patients (97.9%). The most common AEs were hypertension (64.6%) and fatigue (64.6%). The most common grade 3/4 AEs were Aspartate transaminase (AST) and/or Alanine transaminase (ALT) increase (8.3%), fatigue (6.2%) and proteinuria (6.2%). No grade 5 treatment-related AEs were reported. The total number of grade 3/4 AEs was higher among patients with a 4W-RDI <70% (p = 0.01).

**Table 5:** Adverse events.

	All patients		4W-RDI≥70%		4W-RDI<70%		p value*	
	Any grade	Grade 3/4	Any grade	Grade 3/4	Any grade	Grade 3/4	Any grade	Grade 3/4
Total AEs	47(97.9%)	18(37.5%)	23(95.8%)	4(16.7%)	24(100.0%)	14(58.3%)	1.00	0.01
Hand-foot-syndrome	22(45.8%)	0(0.0%)	12(50.0%)	0(0.0%)	10(41.7%)	0(0%)	0.77	-
Diarrhea	10(20.8%)	0(0.0%)	5(20.8%)	0(0.0%)	5(20.8%)	0(0.0%)	1.00	-
Hypertension	31(64.6%)	1(2.1%)	15(62.5%)	0(0.0%)	16(66.7%)	1(4.2%)	1.00	1.00
Decreased appetite	24(50.0%)	2(4.2%)	9(37.5%)	1(4.2%)	15(62.5%)	1(4.2%)	0.15	1.00
Fatigue	31(64.6%)	3(6.2%)	13(54.2%)	0(0.0%)	18(75.0%)	3(12.5%)	0.23	0.23
Proteinuria	19(39.6%)	3(6.2%)	8(33.3%)	0(0.0%)	11(45.8%)	3(12.5%)	0.56	0.23
Nausea	14(29.2%)	2(4.2%)	5(20.8%)	1(4.2%)	9(37.5%)	1(4.2%)	0.34	1.00
Hypothyroidism	16(33.3%)	0(0.0%)	12(50.0%)	0(0.0%)	4(16.7%)	0(0.0%)	0.03	-
AST/ALT increase	20(41.7%)	4(8.3%)	7(29.2%)	0(0.0%)	13(54.2%)	4(16.7%)	0.14	0.11
Others	9(18.8%)	5(10.4%)	3(12.5%)	2(8.3%)	6(25.0%)	3(12.5%)	0.46	1.00

AEs: adverse events; ALT: alanine transaminase; AST: aspartate transaminase; 4W-RDI: 4 week relative dose intensity;

*comparison between 4W-RDI≥70% and 4W-RDI<70%.

## Discussion

This study has shown the importance of maintaining high dose intensity within the initial 4 weeks of LEN therapy to obtain the radiological best response and longer OS. The RDI has been reported to be associated with favorable treatment response to chemotherapy in various cancers, including the use of sorafenib for renal cell carcinoma [11] and regorafenib for colorectal cancer [13]. In this study, we focused on dose intensity of early phase of LEN therapy and found that patients with 4W-RDI ≥70% had better radiological response and longer OS. Although drug interruption or dose modification is often necessary during TKI therapy, a 4W-RDI ≥70% in lenvatinib may serve as a benchmark for obtaining favorable treatment effects.

The relation between radiographic best response and LEN dose was also shown in this study. Patients with 4W-RDI ≥70% showed superior radiological best response rate, that response according to the mRECIST criteria may be dose-dependent. Given that sub-analysis of a phase 3 trial revealed that patients with radiological response have a better OS [21], this association between the 4W-RDI and radiographic response may explain the superior OS in patients with a 4W-RDI ≥70%. The role of 4W-RDI ≥70% as a prognostic factor was also clearly shown by its association with patients being able to continue LEN therapy for longer. Patients with a 4W-RDI ≥70% also had fewer grade 3/4 AEs, indicating that a high 4W-RDI may reflect better tolerability beyond 4 weeks. Longer durations of LEN therapy are expected to maintain the anti-tumor effects for longer and to contribute to longer OS. Finally, it has been reported that the modified ALBI grade is a useful prognostic factor in lenvatinib therapy [22]. In the present study, we revealed that the 4W-RDI was associated with the ALBI score. The higher dose intensity in patients with favorable ALBI grade may be one mechanism underlining the ALBI grade as a prognostic factor of LEN therapy. Also we revealed that 4W-RDI <70% were associated with lower ALBI score and higher rate of severe AEs. Previous reports showed PK of LEN were affected by liver functions [6, 7, 23]. Patients with 4W-RDI <70% and low liver function may be exposed high blood concentration of LEN and suffered severe AEs. Further study is necessary in this point.

This study has some limitations. This study is single center retrospective study and the number of patients was relatively small. Validation in a larger population may be necessary to confirm these findings in the future.

In conclusion, the RDI ≥70% during the initial 4 weeks of lenvatinib therapy is associated with a favorable radiological response and longer OS. Therefore, adequate management of AEs and encouragement of patients to continue therapy at levels exceeding the RDI of ≥70% in the initial 4 weeks of therapy may improve outcomes.

## Supporting information

S1 Table Univariable and multivariable analysis of prognostic factors for survival. (DOCX)

S1 Fig Flow chart of the study cohort. (TIF)
