# Supplementary material for: Relative dose intensity over the first four weeks of lenvatinib therapy is a factor of favorable response and overall survival in patients with unresectable hepatocellular carcinoma
Source: PLoS One. 2020 Apr 20;15(4):e0231828. doi: 10.1371/journal.pone.0231828 (PMC7170221; doi:10.1371/journal.pone.0231828)
Supplement: S1 Table — (DOCX) [file pone.0231828.s001.docx]

S1 Table. Univariable and multivariable analysis of prognostic factors for survival.

|  | | | | | | |
| --- | --- | --- | --- | --- | --- | --- |
|  | Univariable analysis | | | Multivariable analysis | | |
|  | HR | 95%CI | p value | HR | 95%CI | p value |
| Age | 0.99 | 0.94-1.04 | 0.66 |  |  |  |
| Male gender | 0.63 | 0.08-5.07 | 0.67 |  |  |  |
| Body　Weight | 0.98 | 0.95-1.02 | 0.42 |  |  |  |
| 4W-RDI 70%≥ | 0.28 | 0.09-0.90 | 0.03 | 0.3 | 0.09-0.96 | 0.04 |
| TKI　naive | 0.58 | 0.20-1.65 | 0.30 |  |  |  |
| AFP level | 1.00 | 0.99-1.00 | 0.61 |  |  |  |
| ALBI　score | 1.80 | 0.56-5.81 | 0.33 |  |  |  |
| Extrahepatic metastasis | 0.86 | 0.30-2.48 | 0.77 |  |  |  |
| BCLC Stage C | 1.34 | 0.42-4.30 | 0.62 |  |  |  |
| Initial dose reduction | 1.06 | 0.33- | 3.39 |  |  |  |

AFP: α-Fetoprotein; BCLC: Barcelona Clinical Liver Cancer; HR: hazard ratio; TKI: tyrosine kinase inhibitor; 4W-RDI :4 week relative dose intensity.
