# Supplementary figures and images for: Relative dose intensity over the first four weeks of lenvatinib therapy is a factor of favorable response and overall survival in patients with unresectable hepatocellular carcinoma
Source: PLoS One. 2020 Apr 20;15(4):e0231828. doi: 10.1371/journal.pone.0231828 (PMC7170221; doi:10.1371/journal.pone.0231828)

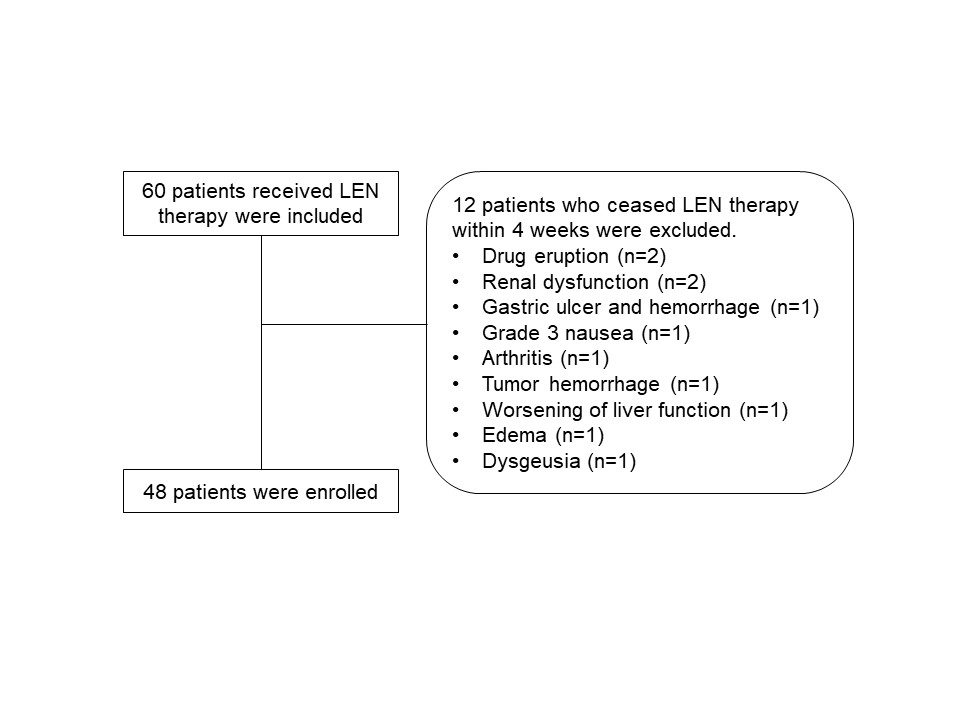

Supplement: S1 Fig — (TIF) [file pone.0231828.s002.tif]
